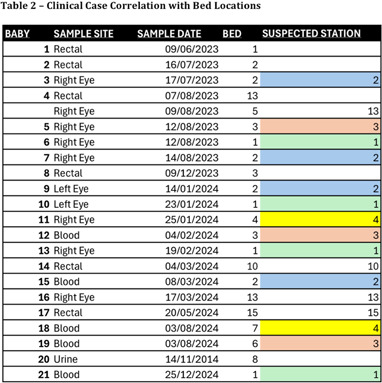# Serratia Outbreak in the (Neonatal Intensive Care Unit (NICU) – Sinking a Stubborn Adversary

**DOI:** 10.1017/ash.2025.368

**Published:** 2025-09-24

**Authors:** Candice Datnow, Jonathan Lellouche, Svetlana Paikin, Aryeh Simmonds, Etty Litig, Marina Afraimov, Danielle Atiya, Talya Finn Fried, Hani Laderman, Moshe Bechor, Vered Schechner

**Affiliations:** 1 Laniado Hospital, Netanya, Israel; 2 Adelson School of Medicine, Ariel University, Ariel, Israel; Sanz Medical Center, Laniado Hospital, Netanaya, Israel; 3 Laniado Hospital, Sanz Medical Center; 4 Laniado Hospital, Adelson School Of Medicine-Ariel University; 5 Laniado Hospital; 6 Sanz Medical Centre; 7 National Institute for Antibiotic Resistance and Infection Control, Ministry of Health, Tel Aviv, Israel; 8 Israel Ministry of Health

## Abstract

**Background:** Serratia marcescens, a recognized environmental pathogen, often contaminates hospital water systems. Infections are typically exogenous, with occasional human reservoirs. NICU outbreaks can result in serious nosocomial infections, including meningitis, bacteremia, and conjunctivitis. Sources include contaminated medical devices, solutions, and hospital water systems, specifically sink traps and outlets, with transmission occurring directly or indirectly via aerosolization. **Outbreak Description:** Between June 2023 and December 2024, an outbreak of S. marcescens occurred in our hospital’s neonatal facility (Figure 1). The facility has 3 main halls - the NICU, intermediate, and “cradle” rooms, along with a breastfeeding, medication, and incubator cleaning room, containing 17 sinks in total (Figures 2 and 3). The outbreak was identified following two Serratia bacteremia cases in early 2024 and a retrospective review revealing 8 positive ocular cultures since mid-2023. After initiating the outbreak investigation, enhancing infection control measures, and conducting engineering repairs, the case rate decreased significantly. However, three additional bacteremia cases, and a urine culture, were subsequently identified. **Infection Control Measures:** Control efforts targeted two reservoirs: patients (via healthcare worker transmission) and the environment. Key measures included reinforcement of hand hygiene, aseptic breastfeeding techniques, contact precautions, and environmental disinfection protocols. Bathing was standardized using sterile water. Environmental Sampling and Investigation: Given Serratia’s known association with waterborne contamination, environmental sampling focused on sink traps and outlets across all areas, revealing persistent contamination despite repeated treatment with concentrated chlorine (Table 1). Epidemiological data identified temporal and spatial correlations between contaminated sinks and clinical cases, notably involving faulty plumbing adjacent to NICU sink 3/4 (Table 2). Water leakage and back pressure from a blocked pipe were hypothesized to cause aerosolization from the connected sink causing infections. Microbiological biotyping clustered clinical and environmental isolates, further implicating aerosolized contamination, including from a sink in the incubator cleaning room used to dispose of hospital wastewater (Figure 4). Outbreak Control: Despite pipe repairs and decontamination, sink contamination recurred due to Serratia’s ability to colonize biofilms in water pipes. Expert consultation emphasized “sink hygiene,” including minimizing equipment storage near sinks, distancing neonates and incubators, and avoiding procedures adjacent to sinks. **Outcome:** This multifactorial approach significantly reduced clinical cases. Continuous environmental monitoring and education aim to eliminate Serratia as a recurring threat in our neonatal facility and broader hospital environment. **Conclusion:** This outbreak highlights the challenges of controlling waterborne pathogens in hospital settings and underscores the importance of combining engineering, environmental decontamination, and behavioral interventions to

**Figure:**
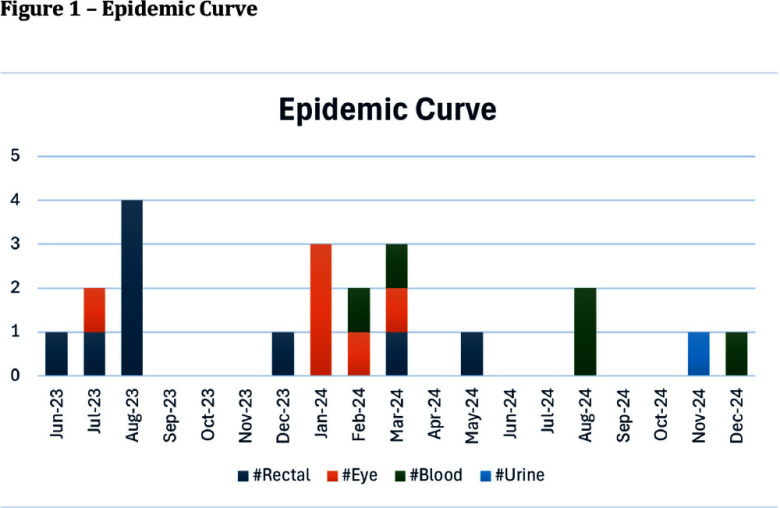

**Figure:**
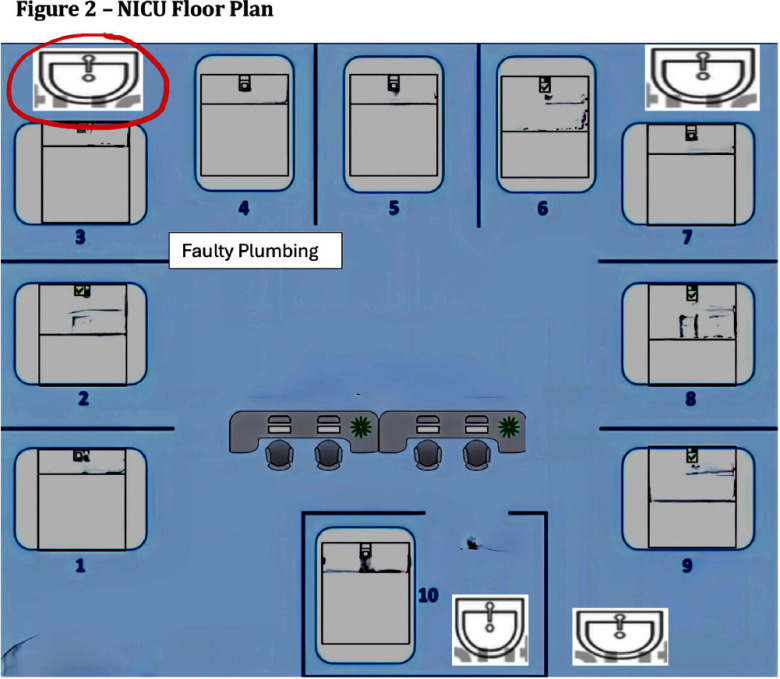

**Figure:**
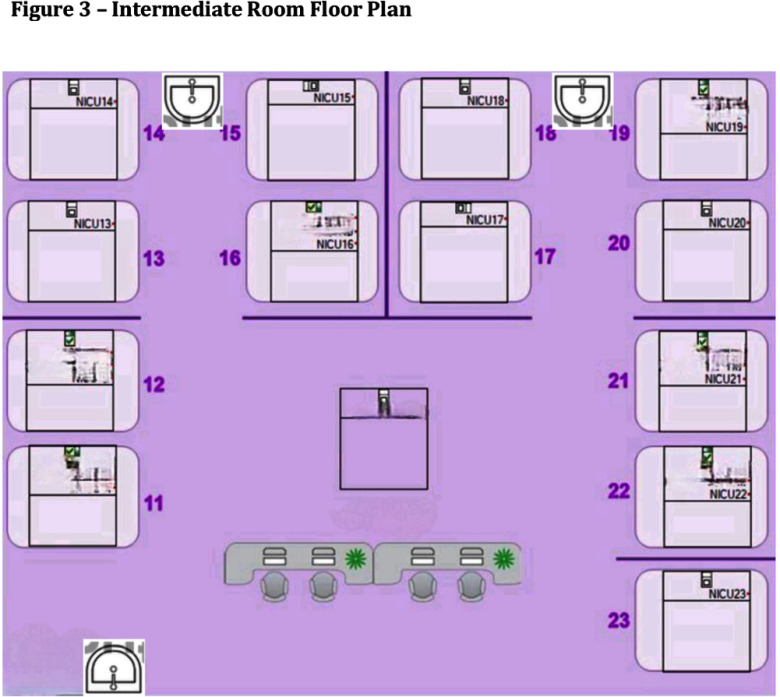

**Figure:**
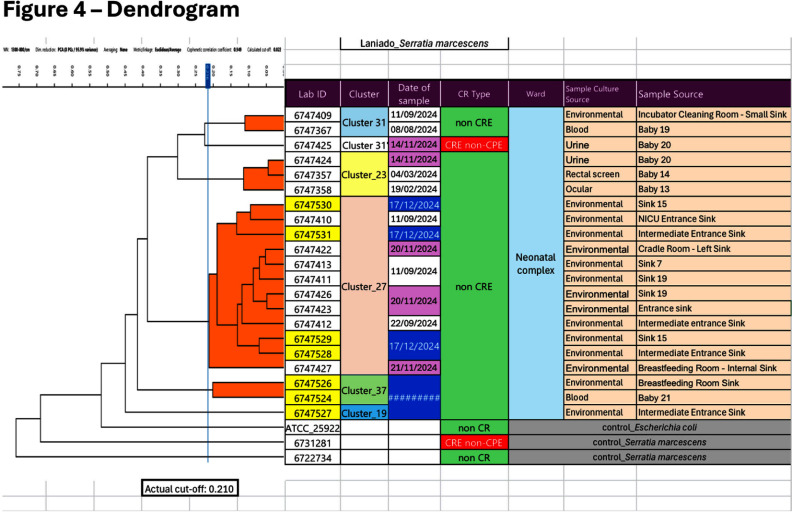

**Figure:**
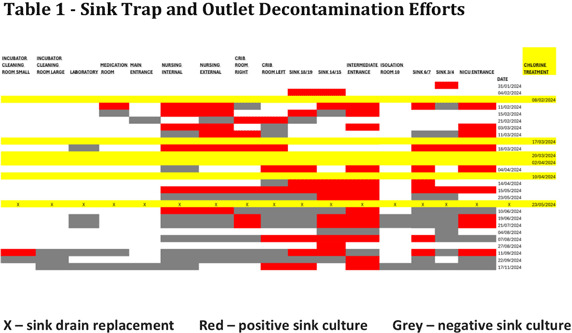

**Figure:**